# Manufacture and Characterization of High Q-Factor Inductors Based on CMOS-MEMS Techniques

**DOI:** 10.3390/s111009798

**Published:** 2011-10-19

**Authors:** Ming-Zhi Yang, Ching-Liang Dai, Jin-Yu Hong

**Affiliations:** Department of Mechanical Engineering, National Chung Hsing University, Taichung, 402, Taiwan; E-Mails: d099061005@mail.nchu.edu.tw (M.-Z.Y.); hunter741213@yahoo.com.tw (J.-Y.H.)

**Keywords:** micro inductors, MEMS, high Q-factor

## Abstract

A high Q-factor (quality-factor) spiral inductor fabricated by the CMOS (complementary metal oxide semiconductor) process and a post-process was investigated. The spiral inductor is manufactured on a silicon substrate. A post-process is used to remove the underlying silicon substrate in order to reduce the substrate loss and to enhance the Q-factor of the inductor. The post-process adopts RIE (reactive ion etching) to etch the sacrificial oxide layer, and then TMAH (tetramethylammonium hydroxide) is employed to remove the silicon substrate for obtaining the suspended spiral inductor. The advantage of this post-processing method is its compatibility with the CMOS process. The performance of the spiral inductor is measured by an Agilent 8510C network analyzer and a Cascade probe station. Experimental results show that the Q-factor and inductance of the spiral inductor are 15 at 15 GHz and 1.8 nH at 1 GHz, respectively.

## 1. Introduction

Micro inductors can be applied in VCO, LC tank and DC-DC converters [1,2]. The Q-factor is an important characteristic for inductors. The energy dissipation in inductors depends on their Q-factor. As the Q-factor of inductors increases, the energy dissipation of inductors decreases. Many studies have utilized MEMS (microelectromechanical system) technology to enhance the Q-factor of micro inductors. For instance, Ahn and Allen [3] fabricated a solenoid inductor with electroplated nickel-iron permalloy cores on silicon wafer using the surface micromachining process. The solenoid inductor had an inductance of 0.1 μH at 10 kHz and a Q-factor of 1.5 at 1 MHz. Nam *et al.* [4] proposed electroplated solenoid-type inductors fabricated on both a standard silicon substrate and glass substrate by thick PR photolithography and copper electroplating. The maximum Q-factor of the inductors was about 10. Chen *et al.* [5] manufactured an edge-suspended inductor using a combination of deep dry etching and anisotropic wet etching techniques. The inductor had an inductance of 4.5 nH, a maximum Q-factor of 11.7 and a self-resonance frequency of 14.3 GHz. Park *et al.* [6] adopted a high-resistivity silicon substrate to improve the Q-factor of micro inductors. The spiral inductors with the rectangular and circular shape were built on the 2 kΩ·cm silicon substrate using the conventional CMOS process without any post-process, and the maximum Q-factor of the inductors was 12. Dai and Tsai [7] presented a micro suspended inductor made by the conventional CMOS process. The suspended inductor was released by a post-process after completion of the CMOS process. The post-process employed a wet etching to etch the sacrificial metal layers, and then TMAH was adopted to remove the underlying silicon substrate. The maximum Q-factor of the inductor was 4.7. Lakdawala *et al.* [8] used the 0.18 μm copper CMOS process and a post-CMOS process to produce a suspended spiral inductor. The post-CMOS process includes an anisotropic RIE CHF_3_/O_2_ dry etching to etch the dielectric layer and an isotropic RIE SF_6_/O_2_ dry etching to remove silicon substrate for releasing the suspended spiral inductor, in which the spiral inductor had a maximum Q-factor of 7.

The technique that uses the commercial CMOS process to manufacture MEMS devices is called CMOS-MEMS [9–11]. Micro devices made by the CMOS-MEMS technique usually need a post-process to release suspended structures [12,13] or to add functional films [14]. The advantages of micro inductors fabricated by this technique include high Q-factor and easy mass-production. In this work, we employ the CMOS-MEMS technique to develop a spiral inductor. In order to enhance the Q-factor of the inductor, a post-process is adopted to remove the underlying silicon substrate. The post-process employs RIE CHF_3_/O_2_ to etch the sacrificial oxide layer, and then TMAH is used to remove the underlying silicon substrate. Experiments indicate that the suspended spiral inductor has a Q-factor of 15 at 15 GHz.

## 2. Structure of the Inductors

**Figure 1.**
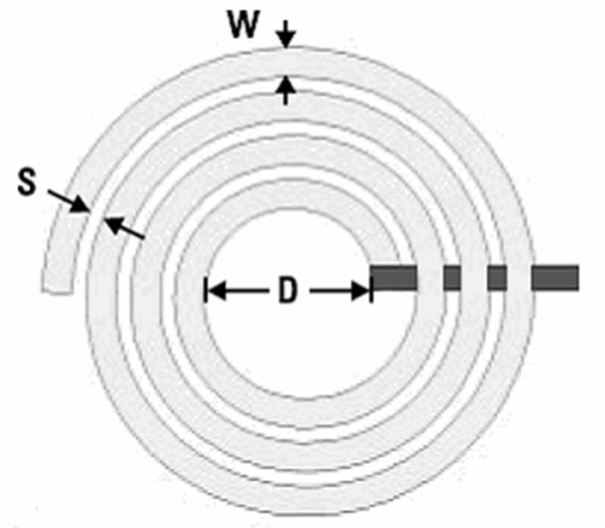
Structure of the spiral inductor.

Figure 1 illustrates a planar spiral inductor, where *W* is the wire width of the spiral inductor, *D* is the internal diameter of the spiral inductor and *S* is the spacing between the wires of the spiral inductor. In this investigation, the planar spiral inductor is designed with *W* = 10 μm, *D* = 136 μm, *S* = 2 μm, its thickness is about 0.95 μm, and the number of turns is 3.5.

**Figure 2.**
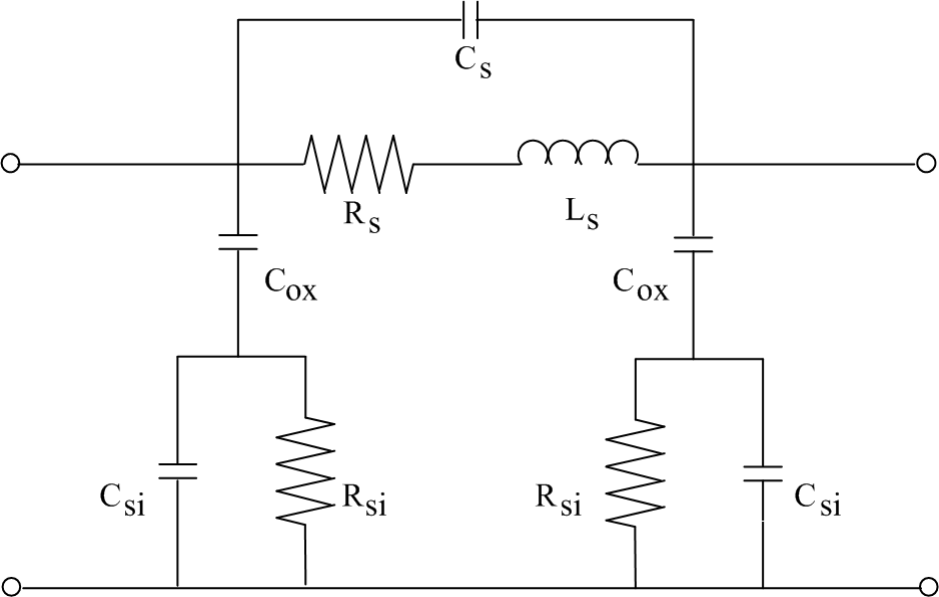
*π* model for the spiral inductor.

The Q-factor of the inductor, which measures the capability of the inductors to save energy, is an important parameter. As shown in Figure 2, the Q-factor of the spiral inductor according to the *π* model is given by [15]:

(1) $$\begin{array}{c} Q=\frac{\omega L_{s}}{R_{s}}\cdot \frac{R_{p}}{R_{p}+[{\left(\omega L_{s}/R_{s}\right)}^{2}+1]R_{s}}\cdot \left[1-\frac{R_{s}^{2}(C_{s}+C_{p})}{L_{s}}-\omega^{2}L_{s}(C_{s}+C_{p})\right]\\ =\frac{\omega L_{s}}{R_{s}}\cdot \left(\begin{array}{l} \text{Substrate loss}\\ \text{factor} \end{array}\right)\cdot \left(\begin{array}{l} \text{Self-resonance}\\ \text{factor} \end{array}\right) \end{array}$$

where:

(2) $$R_{p}=\frac{1}{\omega^{2}C_{\mathrm{ox}}^{2}R_{\mathrm{Si}}}+\frac{R_{\mathrm{Si}}{\left(C_{\mathrm{ox}}+C_{\mathrm{Si}}\right)}^{2}}{C_{\mathrm{ox}}^{2}}$$

(3) $$C_{p}=C_{\mathrm{ox}}\cdot \frac{1+\omega^{2}(C_{\mathrm{ox}}+C_{\mathrm{Si}})C_{\mathrm{Si}}R_{\mathrm{Si}}^{2}}{1+\omega^{2}{\left(C_{\mathrm{ox}}+C_{\mathrm{Si}}\right)}^{2}R_{\mathrm{Si}}^{2}}$$

and *ω* represents frequency; *L_s_* and *R_s_* are the series inductance and resistance, respectively; *C_s_* is the overlap capacitance between the metal lines and the underlying bottom lead wire; *C_ox_* is the dielectric capacitance between the inductors and the substrate; *C_Si_* and *R_Si_* are the silicon substrate capacitance and resistance, respectively. According to Equation (1), we know that the Q-factor of the inductor depends on the substrate loss. The second term in Equation (1) is the substrate loss factor representing the energy dissipated in the silicon substrate. The silicon substrate resistance or the distance between the inductor and the subtrate increase, then the resistance *R_si_* becomes large. Suppose the resistance *R_si_* increases to infinity, the limit of *R_p_* in Equation (2), lim_*R*_*Si* → ∞__ *R_p_* = ∞, becomes infinity. Then, substituting the infinite *R_p_* into the second term in Equation (1), the limit of the substrate loss factor is given by:

(4) $$\text{Substrate loss factor}={\text{lim}}_{R_{p}\to \infty }\frac{R_{p}}{R_{p}+\left[{\left(\frac{\omega L_{s}}{R_{s}}\right)}^{2}+1\right]R_{s}}=1$$

Because the numerator is less than the denominator in the substrate loss factor, this means that the substrate loss factor is less than or equals unity. According to Equation (4), we know that the substrate loss factor is unity as *R_p_* increases infinity, so the substrate loss factor rises as the substrate resistance *R_si_* increases. Therefore, the substrate loss of the inductor can be improved in two ways. One is to increase the resistivity of the silicon substrate to prevent the loss of current in the silicon substrate [6], and the other is to increase the distance between the inductor and silicon substrate surface [16]. In this work, we adopt to increase the distance between the inductor and silicon substrate surface reducing the substrate loss. The underlying silicon substrate is removed by a post-CMOS process to increase this distance.

## 3. Fabrication of the Inductors

**Figure 3.**
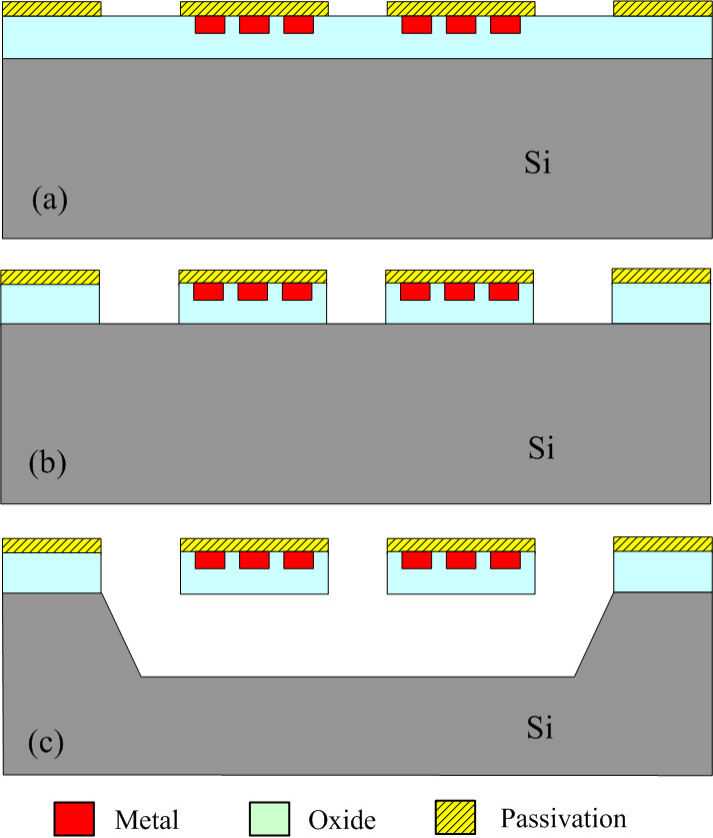
Process flow of the spiral inductor; **(a)** after the CMOS process, **(b)** etching the sacrificial oxide layer, **(c)** removing the underlying silicon substrate.

The commercial TSMC (Taiwan Semiconductor Manufacturing Company) 0.35 μm CMOS process is utilized to manufacture the micro spiral inductor on silicon substrate. The 0.35 μm CMOS process contains one polysilicon layer and four metal layers, in which all metal layers are aluminum and are insulated by silicon dioxide layers. Thickness of each silicon dioxide layer is about 1 μm. The silicon substrate is p-type (1 0 0) orientation. We design the layout of the spiral inductor, and TSMC uses the 0.35 μm CMOS process to fabricate the spiral inductor. Figure 3 shows the process flow of the spiral inductor [17]. Figure 3(a) presents the spiral inductor after completion of the CMOS process. Material of the spiral inductor is aluminum metal. The spiral inductor requires a post-process to remove the underlying silicon substrate in order to reduce the substrate loss and enhance the Q-factor.

**Figure 4.**
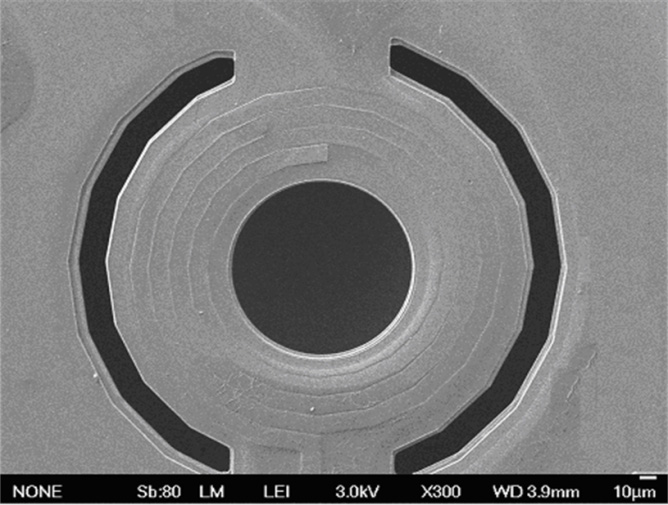
SEM image of the suspended spiral inductor after the post-process.

The post-process includes two steps. One removes the sacrificial oxide layer, and the other step is to etch silicon substrate. Figure 3(b) illustrates the sacrificial layer of silicon dioxide etched by a dry etching. An anisotropic RIE CHF_3_/O_2_ dry etching is employed to etch the sacrificial oxide layer, and to expose silicon substrate. The etching conditions are RF power 150 W, pressure 10 mtorr, gas flow of CHF_3_ 16.8 sccm with O_2_ 4 sccm, and the etching rate is about 900 Å/min. Figure 3(c) depicts that a wet etching is utilized to etch the underlying silicon substrate. The etchant of 25 wt% TMAH at the temperature of 70 °C is used to etch the underlying silicon substrate, and to obtain the suspended spiral inductor. A magnetic stirrer rotating with speed of 100 rpm is utilized to facilitate uniform etching during etching the silicon substrate. The etching rate of the TMAH etchant is about 18 m/h. Figure 4 shows an SEM (scanning electron microscope) image of the spiral inductor after the post-process. The post-process is compatible with the CMOS process.

## 4. Results and Discussion

**Figure 5.**
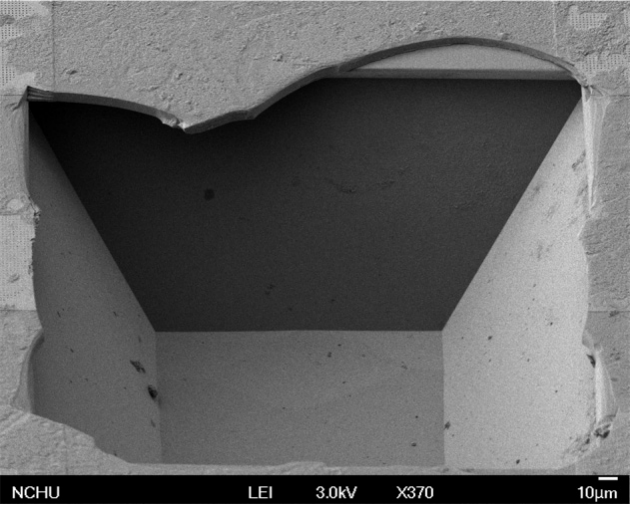
SEM image of the cavity after scraping the suspended spiral inductor off.

A probe was utilized to scrape the suspended spiral inductor for measuring the etching depth between the spiral inductor and silicon substrate after the post-process. Figure 5 shows a SEM image of the cavity on the underlying silicon substrate after scraping the suspended spiral inductor off. A white light interferometer (Zoomsurf 3D from Fogale Nanotech Co.) was used to measure the depth of the cavity.

**Figure 6.**
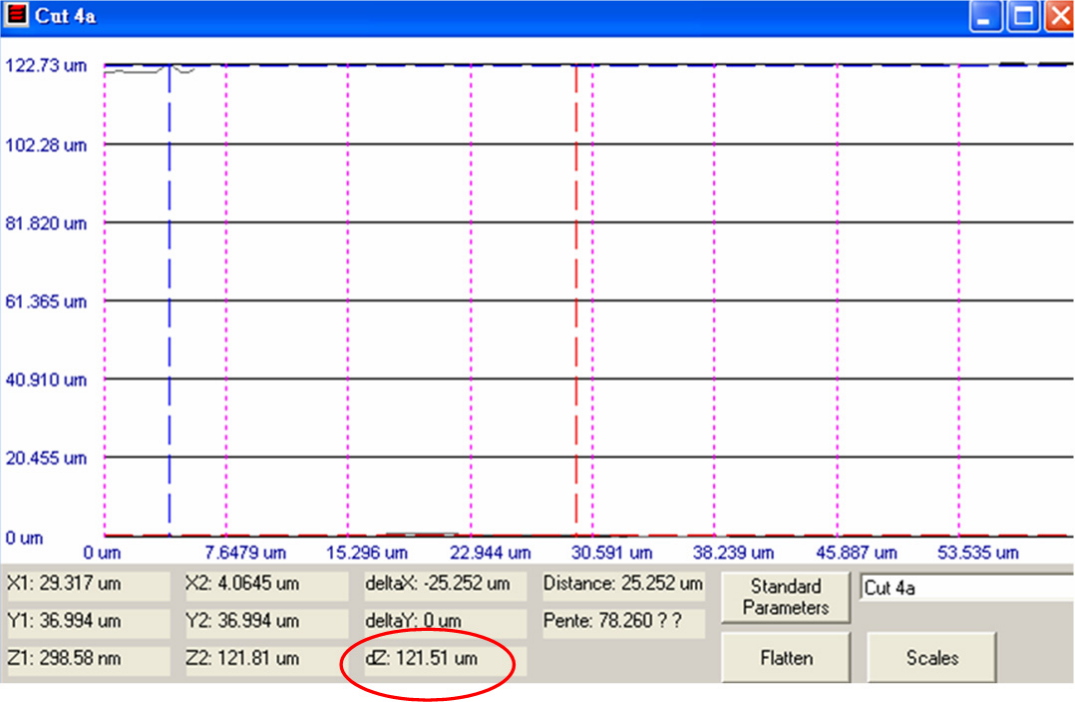
Depth of the cavity measured by a white light interferometer.

Figure 6 shows the measured results of the cavity depth by the white light interferometer. The results revealed that the distance between the spiral inductor and silicon substrate was about 121 μm.

**Figure 7.**
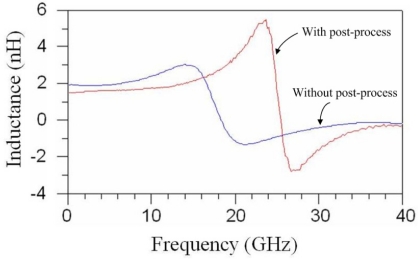
Inductance of the spiral inductor.

The performance of the spiral inductor was measured by an Agilent 8510C network analyzer and a Cascade probe station. The spiral inductor was set on the Cascade probe station, and the network analyzer recorded the inductance and Q-factor of the inductor. The parasitic effect of the pad in the inductor must to be removed by using the de-embedding procedure. Hence, a dummy open pad was designed for de-embedding the parasitic effect. The de-embedding procedure was the measured values of the spiral inductor to subtract the measured values of the dummy open pad to remove the parasitic effect of the cables and the chip. The spiral inductor before and after the post-process was measured in the frequency range of 0.1–40 GHz. Figure 7 displays the inductance of the spiral inductor with and without the post-process.

As shown in Figure 7, the inductance of the inductor without the post-process changed from 2 nH to 3 nH at 0.1–14 GHz, and the inductance of the inductor with the post-process increased from 1.8 nH to 5.5 nH at 0.1–24 GHz. The inductor without the post-process had a self-resonance frequency of about 18 GHz, and the self-resonance frequency of the inductor with the post-process increased to 25.5 GHz.

**Figure 8.**
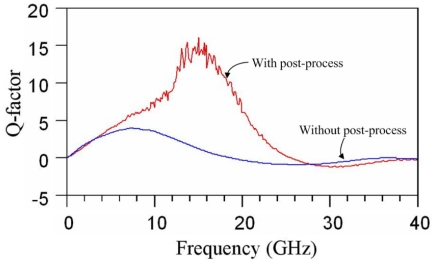
Q-factor of the spiral inductor.

Figure 8 presents the Q-factor of the spiral inductor after de-embedding procedure. As shown in Figure 8, the inductor without the post-process had a maximum Q-factor of 3.7 at 7 GHz, and the maximum Q-factor of the inductor with the post-process was 15 at 15 GHz. The measured results depicted that the maximum Q-factor of the inductor increased from 3.7 to 15 through the pos-process. The results proved that the underlying silicon substrate was removed resulting in the Q-factor of the inductor increased. Therefore, the spiral inductor with the post-process had a Q-factor of 15 at 15 GHz and a self-resonance frequency of 25.5 GHz.

Park *et al.* [6] developed a spiral inductor on a high-resistivity silicon substrate using the conventional CMOS process without any post-process, and the inductors was a maximum Q-factor of 12. Dai and Tsai [7] employed the commercial 0.35 μm CMOS process and a post-process to produce a suspended inductor, and the maximum Q-factor of the suspended inductor was 4.7. Lakdawala *et al.* [8] proposed a suspended spiral inductor manufactured by the 0.18 μm copper CMOS process and a post-process, and the post-process utilized an RIE dry etching to etch silicon substrate to release the suspended spiral inductor, in which the maximum Q-factor of the spiral inductor was 7. Ozgur *et al.* [18] fabricated a spiral inductor on the suspended membrane using the 1.2 μm CMOS process and a post-process. The post-process adopted an isotropic etching to etch the backside of silicon substrate to form the suspended inductor, and the maximum Q-factor of the inductor was 10.5. In this work, the maximum Q-factor of the inductor was 15. A comparison with the literature indicates that the maximum Q-factor of this work exceeds that of Park *et al.* [6], Dai and Tsai [7], Lakdawala *et al.* [8] and Ozgur *et al.* [17].

## 5. Conclusions

The high Q-factor suspended spiral inductor fabricated using the 0.35 μm CMOS process and a post-process has been implemented. In order to reduce the substrate loss and to enhance the Q-factor, the spiral inductor needed a post-process to remove the underlying silicon substrate. The post-process consisted of two steps. One adopted an anisotropic RIE CHF_3_/O_2_ dry etching to remove the sacrificial layer of silicon dioxide for exposing silicon substrate, and the other step was to apply the etchant of TMAH at the temperature of 70 °C to etch the underlying silicon substrate for releasing the suspended spiral inductor. Experimental results revealed that the maximum Q-factor and self-resonance frequency of the spiral inductor were 15 and 25.5 GHz, respectively. The maximum Q-factor of this work exceeded that of Park *et al.* [6], Dai and Tsai [7], and Lakdawala *et al.* [8]. The post-process was compatible with the CMOS process. Therefore, the suspended inductor had a potential for integration with radio-frequency (RF) integrated circuits on-a-chip.
